# Couple-level dyslipidemia and embryological and cumulative pregnancy outcomes in IVF/ICSI cycles: a single-center retrospective cohort study

**DOI:** 10.3389/fendo.2026.1796100

**Published:** 2026-06-18

**Authors:** Ruobing Lei, Shuyi Chen, Weihong Li

**Affiliations:** Reproductive Medical Center, The First Affiliated Hospital of Chongqing Medical University, Chongqing, China

**Keywords:** couple-level metabolic status, cumulative pregnancy outcomes, dyslipidemia, embryological outcomes, infertility, IVF/ICSI

## Abstract

**Background:**

Dyslipidemia is a metabolic factor associated with reproductive outcomes, but evidence on couple-level dyslipidemia in IVF/ICSI remains limited.

**Objective:**

To investigate associations of dyslipidemia in both partners with embryological and cumulative pregnancy outcomes in couples undergoing IVF/ICSI.

**Methods:**

This single-center retrospective cohort study included 209 infertile couples undergoing their first IVF/ICSI cycle between October 2022 and October 2025. Of these, 163 underwent fresh embryo transfer and were included in analyses of ovarian stimulation, embryological, and fresh-cycle outcomes. Cumulative outcomes were evaluated in all 209 couples using fresh and subsequent frozen–thawed embryo transfer cycles derived from the same ovarian stimulation cycle. Dyslipidemia was assessed in both partners, and couples were classified as bilateral normolipidemia, unilateral dyslipidemia, or bilateral dyslipidemia. Multivariable logistic regression and restricted cubic spline analyses were performed.

**Results:**

Women with dyslipidemia had longer stimulation duration, higher total gonadotropin dose, and a lower two-pronuclear (2PN) fertilization rate. Male dyslipidemia was associated with fewer 2PN zygotes, fewer transferable embryos, and a lower 2PN fertilization rate. At the couple level, bilateral dyslipidemia appeared to be associated with less favorable embryological outcomes. After adjustment for female age, female BMI category, male BMI, and cumulative number of embryos transferred, female dyslipidemia was associated with higher odds of cumulative miscarriage (aOR = 4.455, 95% CI: 1.759–11.283) and lower odds of cumulative live birth (aOR = 0.335, 95% CI: 0.164–0.683). Male dyslipidemia was associated with lower odds of cumulative live birth (aOR = 0.309, 95% CI: 0.127–0.751). Compared with bilateral normolipidemia, bilateral dyslipidemia was associated with higher odds of cumulative miscarriage (aOR = 10.579, 95% CI: 1.830–61.174) and lower odds of cumulative live birth (aOR = 0.116, 95% CI: 0.032–0.414). RCS analyses suggested potential association patterns between selected lipid parameters and cumulative outcomes.

**Conclusions:**

Dyslipidemia in either or both partners was associated with less favorable embryological and cumulative pregnancy outcomes in IVF/ICSI cycles. Couples with bilateral dyslipidemia appeared to show less favorable outcome patterns, although these findings should be interpreted cautiously given the retrospective design and limited event numbers.

## 1 Introduction

Infertility is a major reproductive health concern worldwide, affecting approximately one in six adults during their lifetime (1). Assisted reproductive technology (ART), particularly *in vitro* fertilization/intracytoplasmic sperm injection (IVF/ICSI), is an important treatment option for infertile couples (2). However, reproductive outcomes remain heterogeneous, and metabolic status has increasingly been recognized as a potentially modifiable factor associated with ART outcomes (3).

Dyslipidemia is characterized by abnormal circulating levels of triglycerides (TG), total cholesterol (TC), low-density lipoprotein cholesterol (LDL-C), and/or high-density lipoprotein cholesterol (HDL-C) (4). Beyond its cardiovascular relevance, lipid metabolism is involved in energy homeostasis, steroid hormone synthesis, oxidative stress, and inflammatory regulation, which may contribute to gamete competence, embryo development, and pregnancy maintenance (5, 6).

Female dyslipidemia or abnormal lipid profiles have been associated with less favorable ovarian response and ART outcomes. Elevated lipid levels have been linked to altered follicular microenvironment, reduced oocyte quality, altered embryo development, and lower pregnancy or live birth rates after IVF/ICSI (5, 7). Mechanistically, lipid metabolic disturbance may affect oocyte competence and early embryonic development through oxidative stress, mitochondrial dysfunction, and inflammatory activation (6). However, these associations are difficult to interpret because dyslipidemia often coexists with other metabolic factors, particularly elevated body mass index (BMI).

Male dyslipidemia or related metabolic abnormalities have also been associated with altered semen parameters, including sperm concentration, motility, morphology, and sperm DNA integrity (8, 9). Disturbed lipid homeostasis may promote oxidative stress in the testis and spermatozoa, alter sperm membrane composition and cytoskeletal function, and influence fertilization potential and early embryonic development (10). Paternal metabolic status may also affect embryo development through mechanisms not fully captured by routine semen analysis, including sperm-borne epigenetic changes (11).

Most existing studies have evaluated lipid abnormalities in either female or male partners separately. However, ART outcomes are determined by both maternal and paternal contributions, and dyslipidemia in both partners may represent a couple-level metabolic burden rather than an isolated female or male factor. Although recent evidence has examined preconception lipid abnormalities in both partners among couples seeking fertility treatment, data specifically addressing couple-level dyslipidemia in IVF/ICSI cycles, particularly in relation to embryological outcomes and cumulative pregnancy outcomes, remain limited (12).

Therefore, this retrospective cohort study aimed to investigate the associations of dyslipidemia in both partners with embryological and cumulative pregnancy outcomes in couples undergoing their first IVF/ICSI cycle.

## 2 Materials and methods

### 2.1 Study design

This was a single-center retrospective cohort study conducted at the Reproductive Medicine Center of the First Affiliated Hospital of Chongqing Medical University. Clinical data were collected from couples who underwent IVF/ICSI treatment between October 2022 and October 2025. The study was conducted in accordance with the Declaration of Helsinki and was approved by the Ethics Committee of the First Affiliated Hospital of Chongqing Medical University (Approval No. K2023-585).

### 2.2 Study participants

A total of 209 infertile couples who underwent their first IVF/ICSI cycle at our center between October 2022 and October 2025 were retrospectively analyzed. All analyses were performed at the couple level, and each included female patient was matched with her corresponding male partner.

Among the 209 couples, 163 underwent fresh embryo transfer and were included in the analyses of ovarian stimulation parameters, embryological outcomes, and fresh-cycle pregnancy outcomes. The remaining 46 couples had fresh embryo transfer canceled due to clinical indications or personal reasons and subsequently underwent frozen–thawed embryo transfer (FET). Cumulative pregnancy outcomes were evaluated in all 209 couples based on all embryo transfer cycles derived from the same ovarian stimulation cycle.

All included couples were undergoing their first IVF/ICSI cycle, met standard indications for assisted reproductive treatment, and completed either a fresh embryo transfer or at least one FET derived from a single ovarian stimulation cycle.

### 2.3 Inclusion criteria

Inclusion criteria were as follows:

1. infertile couples undergoing their first IVF/ICSI treatment cycle at our center.
2. availability of baseline lipid profiles for both female and male partners before the initiation of ovarian stimulation.
3. use of conventional IVF or ICSI fertilization.
4. completion of fresh embryo transfer or at least one frozen–thawed embryo transfer derived from the same ovarian stimulation cycle.

Infertility etiologies included tubal factor infertility, ovulatory disorders, male factor infertility, immunological infertility, unexplained infertility, and other indications for assisted reproductive treatment.

### 2.4 Exclusion criteria

1. chromosomal abnormalities in either partner.
2. severe systemic diseases or active infectious diseases in either partner, such as systemic lupus erythematosus, active hepatitis B infection, or severe anemia.
3. pelvic tuberculosis or congenital uterine malformations in female partners.
4. azoospermia or severe asthenozoospermia in male partners.
5. malignant tumors in either partner.

Azoospermia and severe asthenozoospermia were excluded because these conditions represent severe male-factor infertility and may substantially affect fertilization method selection, fertilization outcomes, and early embryo development, thereby introducing major confounding in the evaluation of lipid-related reproductive outcomes.

### 2.5 Baseline hormonal and ovarian reserve assessment

Baseline endocrine and ovarian reserve parameters were assessed before the initiation of ovarian stimulation according to standard clinical protocols. Serum follicle-stimulating hormone (FSH), luteinizing hormone (LH), estradiol (E2), and prolactin (PRL) levels were measured on menstrual cycle days 2–3. Anti-Müllerian hormone (AMH) was recorded as a marker of ovarian reserve. Transvaginal sonography (TVS) was performed during the early follicular phase to assess and record the bilateral antral follicle count (AFC).

### 2.6 Assessment of lipid metabolism parameters

To ensure the accuracy and reliability of lipid measurements, serum lipid profiles were obtained from routine pre-treatment clinical assessments before the initiation of ovarian stimulation and were retrospectively extracted from the medical records. All participants underwent blood sampling after an overnight fast. Serum lipid profiles included total cholesterol (TC), triglycerides (TG), low-density lipoprotein cholesterol (LDL-C), and high-density lipoprotein cholesterol (HDL-C).

Dyslipidemia was defined according to the 2016 Chinese Guidelines for the Prevention and Treatment of Dyslipidemia in Adults (4), with diagnostic thresholds of TC ≥ 5.2 mmol/L, TG ≥ 1.7 mmol/L, LDL-C ≥ 3.4 mmol/L, or HDL-C < 1.0 mmol/L. Participants were classified as having dyslipidemia if any one of these criteria was met.

### 2.7 Ovarian stimulation and embryo transfer

Ovarian stimulation protocols were selected individually by clinicians according to routine clinical practice, based on female age, BMI, ovarian reserve markers, baseline endocrine parameters, infertility etiology, previous medical history, and clinical indications. The protocols included the long gonadotropin-releasing hormone (GnRH) agonist protocol, the GnRH antagonist protocol, and the progestin-primed ovarian stimulation (PPOS) protocol. Because stimulation protocols were not assigned for research purposes, protocol selection reflected individualized clinical decision-making rather than random allocation. Therefore, comparisons involving ovarian stimulation parameters, including gonadotropin duration and total gonadotropin dose, were interpreted descriptively.

During ovarian stimulation, patients were monitored every 2–3 days by serum hormone measurements, including estradiol, luteinizing hormone, and progesterone, together with transvaginal ultrasound assessment of follicular development. Gonadotropin dose adjustment was performed according to follicular growth, serum estradiol levels, ovarian response, and the potential risk of ovarian hyperstimulation syndrome. Final oocyte maturation was triggered according to the institutional protocol and individualized clinical assessment, based on follicular development, serum estradiol levels, and ovarian response. In general, triggering was performed when at least one leading follicle reached a mean diameter of ≥16 mm and the serum estradiol level was approximately ≥200 pg/mL per mature follicle. Oocyte retrieval was performed approximately 36 hours after triggering by ultrasound-guided transvaginal aspiration.

Semen analysis was conducted on the day of oocyte retrieval to determine whether conventional IVF or ICSI would be performed. Fertilization outcomes were assessed thereafter, and fresh embryo transfer was performed on day 3 or day 5 after fertilization. Embryo quality was assessed using standard morphological criteria. Cleavage-stage embryos were evaluated according to blastomere number, degree of fragmentation, and blastomere symmetry. Blastocyst quality was assessed using the Gardner grading system. The decision to perform day 3 or day 5 embryo transfer was based on embryo development, the number and quality of available embryos, blastocyst culture strategy, and clinical considerations. Fresh embryo transfer typically involved two good-quality day 3 embryos or one good-quality day 5 blastocyst.

Fresh embryo transfer was canceled in cases of ovarian hyperstimulation syndrome (OHSS) risk, unfavorable endometrial conditions, or other clinical or personal reasons, and these patients proceeded with frozen–thawed embryo transfer in a subsequent cycle. Serum human chorionic gonadotropin (hCG) levels were measured 14 days after embryo transfer. Biochemical pregnancy was defined as an hCG level ≥25 U/L. Transvaginal ultrasound was performed 30 days after transfer, and clinical pregnancy was confirmed by the presence of an intrauterine gestational sac with fetal cardiac activity.

### 2.8 Recorded variables and outcome measures

The following variables were recorded.

1. Baseline characteristics: female and male age, body mass index (BMI), duration of infertility, infertility etiology, AFC, baseline FSH, LH, E2, PRL, AMH, and lipid profiles, including TC, TG, HDL-C, and LDL-C.
2. Ovarian stimulation parameters: total gonadotropin (Gn) dose and duration of Gn administration.
3. Semen and embryological parameters: sperm concentration, progressive motility, and normal sperm morphology; number of retrieved oocytes, metaphase II (MII) oocytes, two-pronuclear (2PN) zygotes, transferable embryos, and good-quality embryos; MII oocyte rate, 2PN fertilization rate, oocyte utilization rate, good-quality embryo rate, and number of embryos transferred.
4. Pregnancy outcomes: fresh-cycle implantation rate, fresh-cycle clinical pregnancy rate, fresh-cycle early miscarriage rate, fresh-cycle live birth rate, cumulative implantation rate, cumulative clinical pregnancy rate, cumulative miscarriage rate, and cumulative live birth rate.

The MII oocyte rate was calculated as the number of MII oocytes divided by the number of retrieved oocytes. The 2PN fertilization rate was calculated as the number of 2PN zygotes divided by the number of inseminated or injected oocytes. The oocyte utilization rate was calculated as the number of transferable embryos divided by the number of retrieved oocytes. The good-quality embryo rate was calculated as the number of good-quality embryos divided by the number of transferable embryos.

The implantation rate was calculated as the number of gestational sacs divided by the number of embryos transferred. The clinical pregnancy rate was calculated as the number of patients with clinical pregnancy divided by the number of patients who underwent embryo transfer. Early miscarriage was defined as pregnancy loss before 12 gestational weeks after clinical pregnancy, and the early miscarriage rate was calculated as the number of early miscarriages divided by the number of clinical pregnancies. The live birth rate was calculated as the number of patients with at least one live birth divided by the number of patients who underwent embryo transfer.

Cumulative pregnancy outcomes were defined by integrating outcomes from all fresh and subsequent frozen–thawed embryo transfer cycles derived from the same ovarian stimulation cycle. Cumulative implantation rate was calculated as the total number of gestational sacs divided by the total number of embryos transferred across all transfer cycles. Cumulative clinical pregnancy rate was calculated as the number of patients who achieved at least one clinical pregnancy divided by the total number of included couples. Cumulative miscarriage rate was calculated as the number of patients who experienced miscarriage divided by the number of patients who achieved cumulative clinical pregnancy. Cumulative live birth rate was calculated as the number of patients who achieved at least one live birth divided by the total number of included couples. Cumulative pregnancy outcomes were assessed up to December 25, 2025. Couples were followed until a live birth occurred, no available embryos remained from the same ovarian stimulation cycle, or the last recorded embryo transfer outcome was available. Couples with ongoing pregnancies at the time of data extraction were not classified as having live birth unless delivery information was available.

### 2.9 Statistical analysis

Statistical analyses were performed using SPSS version 27.0 (IBM Corp., Armonk, NY, USA), GraphPad Prism version 10.4.2 (GraphPad Software, San Diego, CA, USA), and R software version 4.4.1. Continuous variables are presented as mean ± standard deviation (SD), and categorical variables are presented as frequencies and percentages. The normality of continuous variables was assessed before analysis. For comparisons between two groups, normally distributed continuous variables were analyzed using independent-samples t tests, whereas non-normally distributed variables were analyzed using the Mann–Whitney U test. For comparisons among three groups, one-way analysis of variance (ANOVA) or the Kruskal–Wallis test was used, as appropriate. Categorical variables were compared using the chi-square test or Fisher’s exact test, as appropriate.

Linear regression analyses were used to evaluate the associations between lipid parameters and key embryological outcomes. Multivariable binary logistic regression analyses were performed to evaluate the associations of individual partner-level and couple-level dyslipidemia status with cumulative miscarriage and cumulative live birth, with bilateral normolipidemia used as the reference group in couple-level models. Logistic regression models were adjusted for female age, female BMI category, male BMI, and cumulative number of embryos transferred. Female BMI was categorized as <25.0 kg/m² and ≥25.0 kg/m² according to the conventional overweight cutoff commonly used in adult and ART-related studies, whereas male BMI was included as a continuous covariate. Cumulative miscarriage analyses were restricted to patients who achieved cumulative clinical pregnancy, whereas cumulative live birth analyses were conducted in the overall cohort. As a sensitivity analysis, the multivariable logistic regression models for cumulative live birth were repeated after excluding couples in which either partner had a BMI ≥30 kg/m².

Restricted cubic spline (RCS) analyses were performed within the multivariable logistic regression framework to explore potential nonlinear association patterns between continuous lipid parameters and cumulative pregnancy outcomes. Each lipid parameter was modeled separately to reduce multicollinearity among lipid variables. RCS models were fitted with three knots placed at the 10th, 50th, and 90th percentiles and adjusted for the same covariates as the logistic regression models. Overall and nonlinear P values were reported. Variance inflation factor (VIF) analysis was performed for each adjusted model to assess multicollinearity among the corresponding lipid parameter and covariates. A VIF value < 5 was considered to indicate no severe multicollinearity. Because this was a retrospective cohort study based on all eligible couples treated during the study period, no formal *a priori* sample size calculation was performed. A two-sided *P* value < 0.05 was considered statistically significant.

## 3 Results

Before outcome analyses, serum lipid profiles and dyslipidemia phenotypes were further characterized according to female, male, and couple-level dyslipidemia status. The distributions of serum TC, TG, HDL-C, LDL-C, and dyslipidemia phenotypes are summarized in **Supplementary Table 1**.

### 3.1 Female dyslipidemia and fresh-cycle IVF/ICSI outcomes

A total of 163 women who underwent fresh embryo transfer were included in this analysis and were stratified into a female dyslipidemia group (n = 45) and a female normolipidemia group (n = 118) according to serum lipid profiles. No significant differences were observed between the two groups in age, duration of infertility, baseline FSH, LH, E2, PRL, AMH, or AFC (all *P* > 0.05). Women with dyslipidemia had higher body weight and BMI than normolipidemic women, while height was lower in the dyslipidemia group (all *P* < 0.05).

Regarding ovarian stimulation parameters, women with dyslipidemia had a longer duration of gonadotropin administration and a higher total gonadotropin dose than normolipidemic women (both *P* < 0.05). Because ovarian stimulation protocols were individualized rather than assigned for research purposes, these stimulation-related findings were interpreted descriptively. No significant differences were observed in the number of retrieved oocytes, MII oocytes, or 2PN zygotes between the two groups (all P > 0.05). However, the 2PN fertilization rate was significantly lower in the dyslipidemia group than in the normolipidemia group (*P* < 0.05).

A total of 259 embryos were transferred in fresh cycles, including 71 embryos in the dyslipidemia group and 188 embryos in the normolipidemia group. Fresh-cycle implantation rate, clinical pregnancy rate, early miscarriage rate, and live birth rate did not differ significantly between the two groups (all *P* > 0.05) (**Table 1**).

**Table 1:** Female baseline characteristics, ovarian stimulation parameters, embryological outcomes, and fresh-cycle pregnancy outcomes according to female dyslipidemia status.

Variable	NLF, n=118	DYSLIP-F, n=45	P value
Age (years)	30.97 ± 3.83	31.49 ± 4.38	0.456
Height (cm)	158.39 ± 5.00	155.73 ± 4.82	0.003**
Weight (kg)	55.95 ± 7.84	59.02 ± 8.59	0.031*
BMI (kg/m2)	22.30 ± 2.95	24.35 ± 3.50	0.001**
Duration of infertility (years)	3.03 ± 2.72	3.64 ± 3.25	0.228
Baseline FSH (mIU/mL)	6.81 ± 3.46	7.15 ± 3.16	0.561
Baseline LH (mIU/mL)	8.47 ± 6.77	8.50 ± 5.64	0.977
Baseline E2 (pg/mL)	105.26 ± 141.81	73.37 ± 70.58	0.059
PRL (ng/mL)	19.42 ± 9.79	18.23 ± 11.46	0.529
AMH (ng/mL)	3.25 ± 2.09	3.50 ± 2.36	0.502
AFC (n)	12.17 ± 6.82	12.36 ± 6.64	0.876
Duration of Gn administration (days)	11.37 ± 2.09	12.47 ± 2.46	0.005**
Total Gn dose (IU)	1933.09 ± 459.64	2176.94 ± 546.89	0.005**
No. of retrieved oocytes (n)	12.71 ± 5.15	12.36 ± 6.02	0.707
No. of MII oocytes (n)	11.12 ± 4.79	10.18 ± 4.93	0.268
No. of 2PN zygotes (n)	9.03 ± 4.30	7.62 ± 4.30	0.064
No. of transferable embryos (n)	5.60 ± 2.83	5.07 ± 3.23	0.301
No. of good-quality embryos (n)	3.08 ± 2.30	2.98 ± 2.51	0.812
MII oocyte rate (%)	87.80 ± 13.27	83.75 ± 16.10	0.103
2PN fertilization rate (%)	71.52 ± 18.21	62.00 ± 18.37	0.003**
Oocyte utilization rate (%)	47.06 ± 19.58	43.95 ± 19.75	0.368
Good-quality embryo rate (%)	56.19 ± 32.37	57.39 ± 27.53	0.826
No. of embryos transferred (n)	1.59 ± 0.49	1.58 ± 0.50	0.859
Embryo implantation rate, n/N (%)	93/188 (49.47%)	27/71 (38.03%)	0.100
Fresh-cycle clinical pregnancy rate, n/N (%)	71/118 (60.17%)	23/45 (51.11%)	0.295
Fresh-cycle early miscarriage rate, n/N (%)	8/71 (11.27%)	2/23 (8.70%)	0.728
Fresh-cycle live birth rate, n/N (%)	63/118 (53.39%)	21/45 (46.67%)	0.443

Data are presented as mean ± SD or n/N (%). Continuous variables were compared using independent-samples t tests or Mann–Whitney U tests, as appropriate. Categorical variables were compared using chi-square tests or Fisher’s exact tests, as appropriate. NLF, normolipidemic women; DYSLIP-F, dyslipidemic women; BMI, body mass index; FSH, follicle-stimulating hormone; LH, luteinizing hormone; E2, estradiol; PRL, prolactin; AMH, anti-Müllerian hormone; AFC, antral follicle count; Gn, gonadotropin; MII, metaphase II; 2PN, two-pronuclear. *P < 0.05; **P < 0.01.

### 3.2 Male dyslipidemia and fresh-cycle IVF/ICSI outcomes

Among the 163 male partners of couples undergoing fresh embryo transfer, 120 were classified as dyslipidemic and 43 as normolipidemic. No significant differences were observed between the two groups in male age, sperm concentration, or normal sperm morphology (all *P* > 0.05). Compared with normolipidemic men, men with dyslipidemia had a significantly higher BMI (*P* < 0.05). Progressive sperm motility also differed between the two groups, with a higher value observed in the dyslipidemia group (*P* < 0.05).

Regarding embryological outcomes, men with dyslipidemia had fewer 2PN zygotes, fewer transferable embryos, and a lower 2PN fertilization rate than normolipidemic men (all *P* < 0.05). No significant differences were observed in the number of good-quality embryos, good-quality embryo rate, or number of embryos transferred between the two groups (all *P* > 0.05).

Fresh-cycle implantation rate, clinical pregnancy rate, early miscarriage rate, and live birth rate did not differ significantly between the two groups (all *P* > 0.05) (**Table 2**).

**Table 2:** Male baseline characteristics, semen parameters, embryological outcomes, and fresh-cycle pregnancy outcomes according to male dyslipidemia status.

Variable	NLM, n=43	DYSLIP-M, n=120	P value
Age (years)	31.95 ± 4.45	33.33 ± 4.51	0.088
BMI (kg/m2)	22.73 ± 2.99	25.93 ± 3.61	<0.001**
Sperm concentration (million/mL)	79.78 ± 54.20	93.75 ± 58.11	0.171
Progressive motility (%)	39.17 ± 15.68	45.31 ± 17.30	0.042*
Normal sperm morphology (%)	7.14 ± 3.69	7.27 ± 2.83	0.816
No. of 2PN zygotes (n)	10.05 ± 4.46	8.13 ± 4.19	0.013*
No. of transferable embryos (n)	6.21 ± 3.64	5.18 ± 2.62	0.050*
No. of good-quality embryos (n)	3.53 ± 2.74	2.88 ± 2.19	0.115
2PN fertilization rate (%)	77.86 ± 13.99	65.68 ± 19.16	<0.001**
Good-quality embryo rate (%)	54.81 ± 27.82	57.13 ± 32.19	0.676
No. of embryos transferred (n)	1.53 ± 0.50	1.61 ± 0.49	0.404
Embryo implantation rate, n/N (%)	31/66 (46.97%)	89/193 (46.11%)	0.904
Fresh-cycle clinical pregnancy rate, n/N (%)	25/43 (58.14%)	69/120 (57.50%)	0.942
Fresh-cycle early miscarriage rate, n/N (%)	1/25 (4.00%)	9/69 (13.04%)	0.209
Fresh-cycle live birth rate, n/N (%)	24/43 (55.81%)	60/120 (50.00%)	0.513

Data are presented as mean ± SD or n/N (%). Continuous variables were compared using independent-samples t tests or Mann–Whitney U tests, as appropriate. Categorical variables were compared using chi-square tests or Fisher’s exact tests, as appropriate. NLM, normolipidemic men; DYSLIP-M, dyslipidemic men; BMI, body mass index; 2PN, two-pronuclear. *P < 0.05; **P < 0.01.

### 3.3 Couple-level dyslipidemia and fresh-cycle IVF/ICSI outcomes

Among the 163 couples undergoing fresh embryo transfer, 24 were classified as bilateral normolipidemic (B-NL), 113 as unilateral dyslipidemic (U-DYS), and 26 as bilateral dyslipidemic (B-DYS). No significant differences were observed among the three groups in the number of good-quality embryos, good-quality embryo rate, or number of embryos transferred (all *P* > 0.05).

Compared with the B-NL group, couples in the U-DYS group had a significantly lower 2PN fertilization rate, whereas couples in the B-DYS group had fewer 2PN zygotes, fewer transferable embryos, and a lower 2PN fertilization rate (all *P* < 0.05). In pairwise comparisons with the U-DYS group, the B-DYS group also showed fewer 2PN zygotes and a lower 2PN fertilization rate (both *P* < 0.05). These findings suggest a less favorable embryological outcome profile among couples with bilateral dyslipidemia.

Fresh-cycle implantation rate, clinical pregnancy rate, early miscarriage rate, and live birth rate did not differ significantly among the three groups (all *P* > 0.05) (**Table 3**).

**Table 3:** Embryological outcomes and fresh-cycle pregnancy outcomes according to couple-level dyslipidemia status.

Variable	B-NL, n=24	U-DYS, n=113	B-DYS, n=26	P value
No. of 2PN zygotes (n)	10.50 ± 4.05	8.79 ± 4.41	6.27 ± 3.18^a,b^	0.002**
No. of transferable embryos (n)	6.13 ± 3.46	5.61 ± 2.90	4.15 ± 2.26^a^	0.035*
No. of good-quality embryos (n)	3.63 ± 2.92	3.02 ± 2.19	2.65 ± 2.46	0.337
2PN fertilization rate (%)	79.89 ± 10.12	70.37 ± 19.02^a^	52.29 ± 11.68^a,b^	<0.001**
Good-quality embryo rate (%)	56.28 ± 30.95	55.63 ± 31.49	60.62 ± 29.89	0.762
No. of embryos transferred (n)	1.50 ± 0.51	1.61 ± 0.49	1.58 ± 0.50	0.605
Embryo implantation rate, n/N (%)	19/36 (52.78%)	86/182 (47.25%)	15/41 (36.59%)	0.328
Fresh-cycle clinical pregnancy rate, n/N (%)	15/24 (62.50%)	66/113 (58.41%)	13/26 (50.00%)	0.644
Fresh-cycle early miscarriage rate, n/N (%)	0/15 (0.00%)	9/66 (13.64%)	1/13 (7.69%)	0.282
Fresh-cycle live birth rate, n/N (%)	15/24 (62.50%)	57/113 (50.44%)	12/26 (46.15%)	0.470

Data are presented as mean ± SD or n/N (%). Continuous variables were compared using one-way ANOVA or Kruskal–Wallis tests, as appropriate. Categorical variables were compared using chi-square tests or Fisher’s exact tests, as appropriate. B-NL, bilateral normolipidemia; U-DYS, unilateral dyslipidemia; B-DYS, bilateral dyslipidemia; 2PN, two-pronuclear. ^a^*P* < 0.05 vs. the B-NL group; ^b^*P* < 0.05 vs. the U-DYS group. *P < 0.05; **P < 0.01.

### 3.4 Associations between lipid profiles and key embryological outcomes in fresh-cycle IVF/ICSI

Linear regression analyses were performed to evaluate the associations between serum lipid parameters and selected embryological outcomes in the fresh-cycle cohort. Serum lipid parameters included TC, TG, HDL-C, and LDL-C.

**Figure 1:**
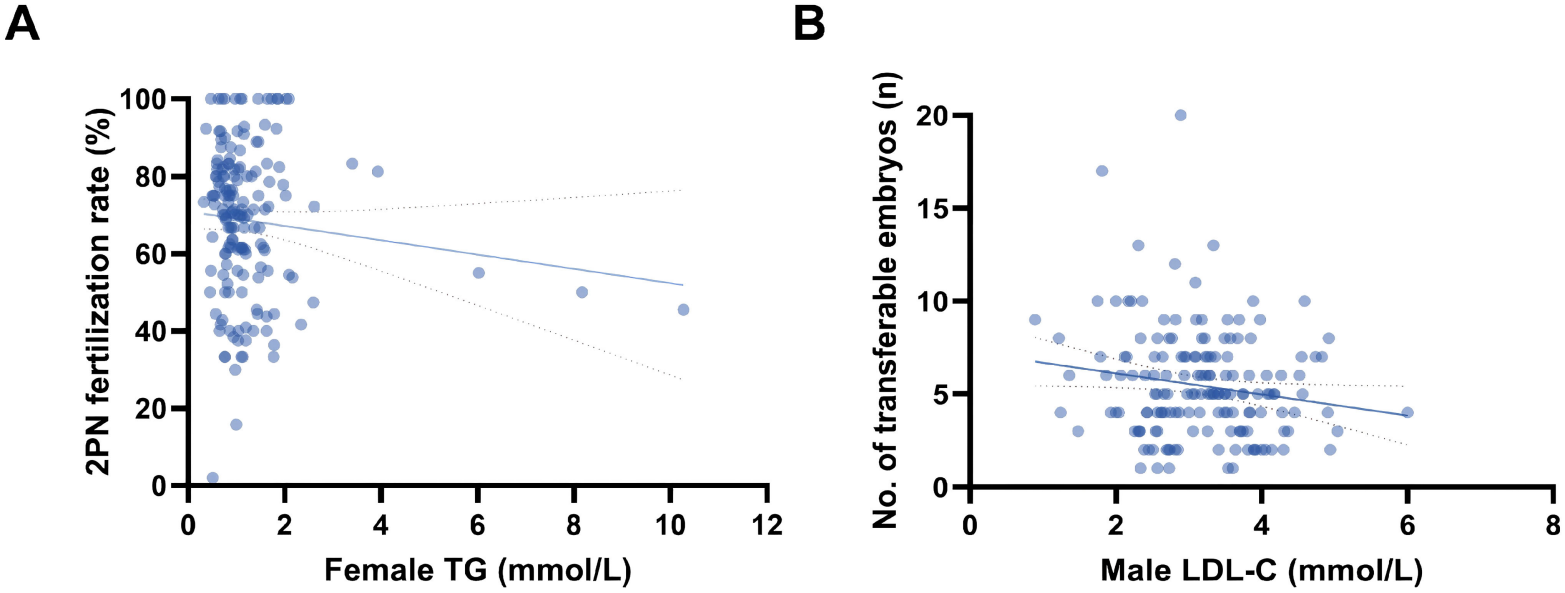
Associations between selected lipid parameters and embryological outcomes in fresh-cycle IVF/ICSI. **(A)** Association between female serum TG level and the 2PN fertilization rate. Higher female TG levels were associated with a lower 2PN fertilization rate (β = −3.148, *P* = 0.017, R² = 0.035). **(B)** Association between male serum LDL-C level and the number of transferable embryos. Higher male LDL-C levels were associated with fewer transferable embryos (β = −0.568, *P* = 0.038, R² = 0.027). The solid lines represent fitted linear regression lines, and the dashed lines indicate 95% confidence intervals.

In the female analysis, serum TG level was negatively associated with the 2PN fertilization rate. Higher serum TG concentrations were associated with a lower 2PN fertilization rate (β = −3.148, *P* = 0.017, R² = 0.035) (**Figure 1A**). The fitted regression equation was as follows:

2PN fertilization rate (%) = 72.821 − 3.148 × TG (mmol/L).

In the male analysis, serum LDL-C level was negatively associated with the number of transferable embryos. Higher serum LDL-C concentrations were associated with fewer transferable embryos (β = −0.568, *P* = 0.038, R² = 0.027) (**Figure 1B**). The fitted regression equation was as follows:

Number of transferable embryos = 7.257 − 0.568 × LDL-C (mmol/L).

### 3.5 Cumulative pregnancy outcomes according to dyslipidemia status

#### 3.5.1 Cumulative pregnancy outcomes according to female dyslipidemia status

A total of 209 couples were included in the cumulative outcome analysis. Among them, 65 female partners were classified as dyslipidemic and underwent the transfer of 136 embryos, whereas 144 female partners were normolipidemic and underwent the transfer of 320 embryos.

Compared with couples with normolipidemic female partners, couples with dyslipidemic female partners had a significantly higher cumulative miscarriage rate and a significantly lower cumulative live birth rate (both *P* < 0.05). No significant differences were observed in cumulative implantation rate or cumulative clinical pregnancy rate between the two groups (**Table 4**).

**Table 4:** Cumulative pregnancy outcomes according to female dyslipidemia status.

Variable	NLF, n=144	DYSLIP-F, n=65	P value
Cumulative implantation rate, n/N (%)	172/320 (53.75%)	65/136 (47.79%)	0.244
Cumulative clinical pregnancy rate, n/N (%)	123/144 (85.42%)	52/65 (80.00%)	0.326
Cumulative miscarriage rate, n/N (%)	17/123 (13.82%)	15/52 (28.85%)	0.019*
Cumulative live birth rate, n/N (%)	111/144 (77.08%)	39/65 (60.00%)	0.011*

Data are presented as n/N (%). Categorical variables were compared using chi-square tests or Fisher’s exact tests, as appropriate. NLF, normolipidemic female partners; DYSLIP-F, dyslipidemic female partners. *P < 0.05.

#### 3.5.2 Cumulative pregnancy outcomes according to male dyslipidemia status

Among the 209 couples included in the cumulative outcome analysis, 147 male partners were classified as dyslipidemic and underwent the transfer of 323 embryos, whereas 62 male partners were normolipidemic and underwent the transfer of 133 embryos.

Compared with couples with normolipidemic male partners, couples with dyslipidemic male partners had a significantly higher cumulative miscarriage rate and a significantly lower cumulative live birth rate (both *P* < 0.05). No significant differences were observed in cumulative implantation rate or cumulative clinical pregnancy rate between the two groups (**Table 5**).

**Table 5:** Cumulative pregnancy outcomes according to male dyslipidemia status.

Variable	NLM, n=62	DYSLIP-M, n=147	P value
Cumulative implantation rate, n/N (%)	72/133 (54.14%)	165/323 (51.08%)	0.553
Cumulative clinical pregnancy rate, n/N (%)	54/62 (87.10%)	121/147 (82.31%)	0.392
Cumulative miscarriage rate, n/N (%)	5/54 (9.26%)	27/121 (22.31%)	0.039*
Cumulative live birth rate, n/N (%)	53/62 (85.48%)	97/147 (65.99%)	0.004*

Data are presented as n/N (%). Categorical variables were compared using chi-square tests or Fisher’s exact tests, as appropriate. NLM, normolipidemic male partners; DYSLIP-M, dyslipidemic male partners. *P < 0.05.

#### 3.5.3 Cumulative pregnancy outcomes according to couple-level dyslipidemia status

Among the 209 couples included in the cumulative outcome analysis, 37 were classified as bilateral normolipidemic (B-NL), 132 as unilateral dyslipidemic (U-DYS), and 40 as bilateral dyslipidemic (B-DYS).

Compared with both the B-NL and U-DYS groups, the B-DYS group had a significantly higher cumulative miscarriage rate and a significantly lower cumulative live birth rate (both *P* < 0.05). No significant differences were observed in cumulative implantation rate or cumulative clinical pregnancy rate among the three groups (**Table 6**).

**Table 6:** Cumulative pregnancy outcomes according to couple-level dyslipidemia status.

Variable	B-NL, n=37	U-DYS, n=132	B-DYS, n=40	P value
Cumulative implantation rate, n/N (%)	45/77 (58.44%)	154/299 (51.51%)	38/80 (47.50%)	0.376
Cumulative clinical pregnancy rate, n/N (%)	33/37 (89.19%)	111/132 (84.09%)	31/40 (77.50%)	0.375
Cumulative miscarriage rate, n/N (%)	2/33 (6.06%)	18/111 (16.22%)	12/31 (38.71%)a,b	0.002**
Cumulative live birth rate, n/N (%)	33/37 (89.19%)	98/132 (74.24%)	19/40 (47.50%)^a^^,^^b^	<0.001**

Data are presented as n/N (%). Categorical variables were compared using chi-square tests or Fisher’s exact tests, as appropriate. B-NL, bilateral normolipidemia; U-DYS, unilateral dyslipidemia; B-DYS, bilateral dyslipidemia.

^a^ *P* < 0.05 vs. the B-NL group;

^b^ *P* < 0.05 vs. the U-DYS group.

**P < 0.01.

### 3.6 Multivariable logistic regression and restricted cubic spline analyses in relation to cumulative pregnancy outcomes

To further evaluate the associations between dyslipidemia status in both partners and cumulative pregnancy outcomes, multivariable binary logistic regression models were constructed. The models were adjusted for female age, female BMI category, male BMI, and the cumulative number of embryos transferred. Female BMI was entered as a categorical covariate using a cutoff of 25.0 kg/m², whereas male BMI was included as a continuous covariate. The analysis of cumulative miscarriage was restricted to patients who achieved cumulative clinical pregnancy, including 175 couples with 32 miscarriage events. The analysis of cumulative live birth was conducted in the overall cohort of 209 couples, including 150 live birth events.

In the multivariable model including dyslipidemia status in both female and male partners, female dyslipidemia was significantly associated with higher odds of cumulative miscarriage (aOR = 4.455, 95% CI: 1.759–11.283, *P* = 0.002) and lower odds of cumulative live birth (aOR = 0.335, 95% CI: 0.164–0.683, *P* = 0.003). Male dyslipidemia was significantly associated with lower odds of cumulative live birth (aOR = 0.309, 95% CI: 0.127–0.751, P = 0.010), whereas its association with cumulative miscarriage did not reach statistical significance (aOR = 2.642, 95% CI: 0.813–8.585, *P* = 0.106) (**Table 7**).

**Table 7:** Multivariable logistic regression analysis of the association between dyslipidemia status in each partner and cumulative pregnancy outcomes.

Outcome	Exposure variable	Reference group	aOR	95% CI	*P* value
Cumulative miscarriage	Female dyslipidemia	Female normolipidemia	4.455	1.759–11.283	0.002
Cumulative miscarriage	Male dyslipidemia	Male normolipidemia	2.642	0.813–8.585	0.106
Cumulative live birth	Female dyslipidemia	Female normolipidemia	0.335	0.164–0.683	0.003
Cumulative live birth	Male dyslipidemia	Male normolipidemia	0.309	0.127–0.751	0.010

aOR, adjusted odds ratio; CI, confidence interval. The model included dyslipidemia status in both female and male partners and was adjusted for female age, female BMI category, male BMI, and cumulative number of embryos transferred. The analysis of cumulative miscarriage was restricted to patients who achieved cumulative clinical pregnancy, whereas the analysis of cumulative live birth included the overall cohort. A two-sided *P* value < 0.05 was considered statistically significant.

After further classification according to couple-level dyslipidemia status, unilateral dyslipidemia was not significantly associated with cumulative miscarriage or cumulative live birth compared with bilateral normolipidemia. In contrast, bilateral dyslipidemia was significantly associated with higher odds of cumulative miscarriage (aOR = 10.579, 95% CI: 1.830–61.174, *P* = 0.008) and lower odds of cumulative live birth (aOR = 0.116, 95% CI: 0.032–0.414, *P* = 0.001) (**Table 8**). These findings suggest that dyslipidemia in both partners may be associated with less favorable cumulative pregnancy outcomes.

**Table 8:** Multivariable logistic regression analysis of the association between couple-level dyslipidemia status and cumulative pregnancy outcomes.

Outcome	Couple-level dyslipidemia status	Reference group	aOR	95% CI	*P* value
Cumulative miscarriage	Unilateral dyslipidemia	Bilateral normolipidemia	2.430	0.478–12.356	0.285
Cumulative miscarriage	Bilateral dyslipidemia	Bilateral normolipidemia	10.579	1.830–61.174	0.008
Cumulative live birth	Unilateral dyslipidemia	Bilateral normolipidemia	0.389	0.124–1.224	0.107
Cumulative live birth	Bilateral dyslipidemia	Bilateral normolipidemia	0.116	0.032–0.414	0.001

aOR, adjusted odds ratio; CI, confidence interval. The model was adjusted for female age, female BMI category, male BMI, and cumulative number of embryos transferred. Bilateral normolipidemia was used as the reference group. The analysis of cumulative miscarriage was restricted to patients who achieved cumulative clinical pregnancy, whereas the analysis of cumulative live birth included the overall cohort. A two-sided *P* value < 0.05 was considered statistically significant.

As a sensitivity analysis, the multivariable logistic regression models for cumulative live birth were repeated after excluding couples in which either partner had a BMI ≥30 kg/m². A total of 184 couples were included in this analysis, with 134 cumulative live birth events. The associations between dyslipidemia status and cumulative live birth were generally consistent with the main analysis. In the partner-level model, female dyslipidemia (aOR = 0.309, 95% CI: 0.142–0.671, P = 0.003) and male dyslipidemia (aOR = 0.302, 95% CI: 0.119–0.767, P = 0.012) remained associated with lower odds of cumulative live birth. In the couple-level model, bilateral dyslipidemia remained associated with lower odds of cumulative live birth (aOR = 0.101, 95% CI: 0.026–0.392, P = 0.001), whereas the association for unilateral dyslipidemia did not reach statistical significance (aOR = 0.361, 95% CI: 0.110–1.188, P = 0.094) (**Supplementary Table 2**).

To further explore potential association patterns between specific lipid parameters and cumulative pregnancy outcomes, restricted cubic spline (RCS) analyses were performed within multivariable logistic regression models. All RCS models were fitted with three knots placed at the 10th, 50th, and 90th percentiles of each lipid parameter and were adjusted for female age, female BMI category, male BMI, and cumulative number of embryos transferred.

To reduce multicollinearity among lipid variables, each lipid parameter was modeled separately in the RCS analyses. VIF analysis was performed for each adjusted model including the corresponding lipid parameter and covariates. All VIF values were below 5, with a maximum VIF of 1.42, suggesting no evidence of severe multicollinearity among the variables included in the adjusted models.

For cumulative miscarriage, female TC, female LDL-C, and male TC showed significant overall associations with the odds of cumulative miscarriage. The tests for nonlinearity were not statistically significant, suggesting approximately linear positive associations. In the corresponding adjusted models, female TC, female LDL-C, and male TC each showed positive associations with the odds of cumulative miscarriage, with the corresponding ORs per 1 mmol/L increase presented in **Table 9**.

**Table 9:** Restricted cubic spline analysis of lipid parameters and cumulative pregnancy outcomes.

Outcome	Lipid parameter	N/events	*P* for overall	*P* for nonlinear	OR per 1 mmol/L	95% CI	*P* for linear trend
Cumulative miscarriage	Female TC	175/32	0.010	0.145	2.490	1.251–4.955	0.009
Cumulative miscarriage	Female LDL-C	175/32	0.043	0.794	2.482	1.201–5.130	0.014
Cumulative miscarriage	Male TC	175/32	0.005	0.059	1.771	1.146–2.737	0.010
Cumulative live birth	Female TG	209/150	0.016	0.039	0.720	0.495–1.048	0.086
Cumulative live birth	Male TC	209/150	0.011	0.091	0.647	0.456–0.916	0.014

RCS, restricted cubic spline; TC, total cholesterol; TG, triglycerides; LDL-C, low-density lipoprotein cholesterol; OR, odds ratio; CI, confidence interval. RCS models were fitted with three knots placed at the 10th, 50th, and 90th percentiles of each lipid parameter. All models were adjusted for female age, female BMI category, male BMI, and cumulative number of embryos transferred. The analysis of cumulative miscarriage was restricted to patients who achieved cumulative clinical pregnancy, whereas the analysis of cumulative live birth included the overall cohort. *P* for overall indicates the overall association between the lipid parameter and the outcome; *P* for nonlinear indicates the test for nonlinearity. For female TG and cumulative live birth, OR per 1 mmol/L should be interpreted as an overall trend estimate because a significant nonlinear association was observed. A two-sided *P* value < 0.05 was considered statistically significant.

**Figure 2:**
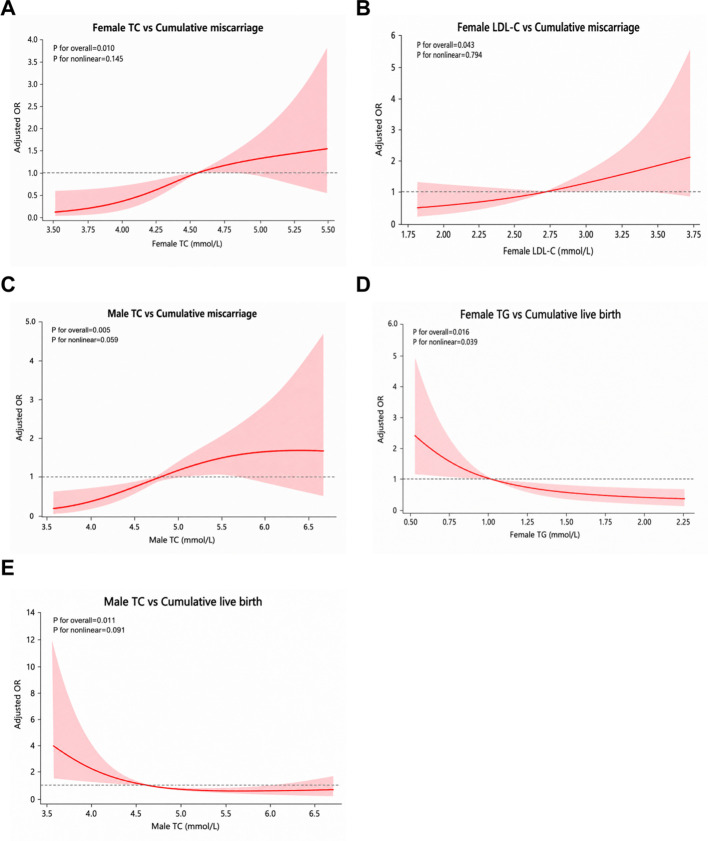
Restricted cubic spline analyses of lipid parameters in relation to cumulative pregnancy outcomes. **(A)** Association between female TC and cumulative miscarriage. **(B)** Association between female LDL-C and cumulative miscarriage. **(C)** Association between male TC and cumulative miscarriage. **(D)** Association between female TG and cumulative live birth. **(E)** Association between male TC and cumulative live birth. The red solid lines represent adjusted odds ratios, the shaded areas represent 95% confidence intervals, and the dashed horizontal lines indicate an odds ratio of 1. All models were adjusted for female age, female BMI category, male BMI, and cumulative number of embryos transferred. TC, total cholesterol; TG, triglycerides; LDL-C, low-density lipoprotein cholesterol; OR, odds ratio; BMI, body mass index.

For cumulative live birth, female TG showed a significant overall association, and the test for nonlinearity was also statistically significant, suggesting a potential nonlinear association pattern. The RCS curve indicated an overall decreasing trend in the odds of cumulative live birth with increasing female TG levels. Male TC was also significantly associated with cumulative live birth, whereas the test for nonlinearity was not statistically significant, suggesting an approximately linear inverse association (**Table 9**; **Figure 2**).

## 4 Discussion

In this single-center retrospective cohort study, we evaluated the associations between dyslipidemia in both partners and IVF/ICSI outcomes, including embryological outcomes in fresh embryo transfer cycles and cumulative pregnancy outcomes derived from a single ovarian stimulation cycle. The main findings were as follows. First, female dyslipidemia was associated with longer gonadotropin administration, higher total gonadotropin dose, and a lower 2PN fertilization rate. Second, male dyslipidemia was associated with fewer 2PN zygotes, fewer transferable embryos, and a lower 2PN fertilization rate. Third, at the couple level, bilateral dyslipidemia was associated with a less favorable embryological outcome profile and less favorable cumulative pregnancy outcomes. In adjusted models, female dyslipidemia was associated with higher odds of cumulative miscarriage and lower odds of cumulative live birth, whereas male dyslipidemia was associated with lower odds of cumulative live birth. Bilateral dyslipidemia was also associated with higher odds of cumulative miscarriage and lower odds of cumulative live birth compared with bilateral normolipidemia.

Female dyslipidemia appeared to be closely linked to a less favorable metabolic and ovarian stimulation profile. In the present study, women with dyslipidemia had higher BMI, longer gonadotropin administration, and higher total gonadotropin dose than normolipidemic women. This finding is biologically plausible, as adiposity, lipid metabolism, and reproductive endocrine function are closely interconnected (3, 13, 14). Excess adiposity and metabolic dysfunction may alter insulin sensitivity, inflammatory status, steroid hormone metabolism, and the follicular microenvironment, thereby influencing ovarian response and treatment requirements during IVF/ICSI cycles. Importantly, these findings should be interpreted in the context of the close relationship between BMI and lipid metabolism. In the present cohort, BMI differed significantly between dyslipidemic and normolipidemic groups, indicating that dyslipidemia may partly reflect a broader metabolic phenotype rather than an isolated lipid abnormality. Although female BMI category and male BMI were adjusted for in the multivariable models, residual confounding related to adiposity, insulin resistance, and other metabolic factors cannot be fully excluded. Therefore, the observed associations should not be interpreted as effects of dyslipidemia completely independent of BMI or adiposity.

At the same time, gonadotropin exposure should be interpreted within the framework of individualized ovarian stimulation. In routine IVF/ICSI practice, protocol selection and gonadotropin dose adjustment are commonly guided by age, BMI, ovarian reserve markers, follicular growth, endocrine response, and the risk of ovarian hyperstimulation (15, 16). In this retrospective cohort, ovarian stimulation protocols were selected according to routine clinical practice rather than assigned for research purposes. Therefore, the longer stimulation duration and higher gonadotropin dose observed in women with dyslipidemia may reflect not only metabolic status but also ovarian reserve, endocrine profile, BMI, and individualized clinical management. Accordingly, these stimulation-related findings should be interpreted as descriptive associations rather than evidence that dyslipidemia directly increases gonadotropin requirements. Previous studies have reported that higher or excessive gonadotropin exposure is associated with embryo development and pregnancy outcomes, although these associations may vary according to ovarian reserve and patient prognosis (17, 18). These findings suggest that female dyslipidemia may be part of a broader metabolic profile relevant to ovarian stimulation and early reproductive outcomes.

Beyond ovarian stimulation, female lipid status was also associated with fertilization-related outcomes. In the present study, female dyslipidemia was associated with a lower 2PN fertilization rate, and serum TG level showed a negative linear association with the 2PN fertilization rate. Although the relatively low R² value indicates that TG explained only a small proportion of the variation in fertilization outcomes, this finding is consistent with the multifactorial nature of oocyte competence and early embryo development. Previous clinical studies have reported associations between abnormal serum lipid profiles and embryo quality, pregnancy outcomes, and cumulative live birth in women undergoing IVF/ICSI or assisted reproduction (5, 7, 19, 20).

The biological basis for this association may involve the follicular microenvironment and oocyte metabolic homeostasis. Lipids are essential substrates for energy production, membrane structure, and steroid hormone synthesis, but excessive lipid accumulation may induce lipotoxic stress and disturb oocyte developmental competence. Experimental and systematic evidence suggests that high-fat diet exposure or lipid metabolic disturbance may impair oocyte and follicular quality through oxidative stress, mitochondrial dysfunction, altered steroidogenesis, meiotic abnormalities, and inflammatory activation (6, 21–23). Together, these findings suggest a biologically plausible association between female lipid disturbance and less favorable fertilization-related outcomes, while also highlighting the multifactorial nature of fertilization outcomes in IVF/ICSI.

Male lipid metabolism also appeared to be associated with embryological outcomes. In the present study, male dyslipidemia was associated with fewer 2PN zygotes, fewer transferable embryos, and a lower 2PN fertilization rate. In addition, male LDL-C level was negatively associated with the number of transferable embryos. These findings are consistent with previous evidence linking dyslipidemia, metabolic syndrome, and lipid-related metabolic abnormalities to altered semen parameters and male infertility (8, 9, 24).

Several biological pathways may help explain this association. Disturbed lipid homeostasis may affect testicular function and spermatogenesis, partly through oxidative stress, endocrine disturbance, and impaired lipid handling within testicular cells (10, 25, 26). Lipids are also critical components of the sperm plasma membrane, and abnormal lipid composition may influence membrane fluidity, capacitation, acrosome reaction, motility, and sperm–oocyte interaction (9, 10). Recent evidence further suggests that lipid stress may affect the sperm cytoskeleton, thereby influencing sperm structure and function (10). In addition, oxidative stress can damage sperm DNA and impair sperm functional competence, while paternal metabolic status may influence early embryonic development through sperm-borne epigenetic changes that are not fully captured by routine semen analysis (11, 27). Therefore, the observed association between male dyslipidemia and less favorable embryological outcomes may reflect not only conventional semen-related parameters but also more subtle sperm functional or molecular alterations. Notably, although progressive motility was higher in men with dyslipidemia in the present cohort, routine semen parameters may not fully capture sperm functional competence, DNA integrity, epigenetic features, or other molecular changes relevant to fertilization and early embryo development.

A notable feature of the present study is the evaluation of dyslipidemia at the couple level. This perspective is biologically relevant because fertilization and early embryo development depend on coordinated maternal and paternal contributions. Successful fertilization requires a series of sperm–oocyte interactions, including sperm capacitation, acrosome exocytosis, sperm–oocyte membrane interaction, oocyte activation, and zygote formation (28). Lipid homeostasis is involved in several of these processes. In spermatozoa, lipid remodeling during acrosome exocytosis, cholesterol efflux, and lipid raft organization are closely related to capacitation, fertilization potential, sperm maturation, and early embryogenesis (29–31). More broadly, lipid signaling participates in gamete maturation, while follicular lipoprotein metabolism and oocyte cholesterol homeostasis have been implicated in oocyte biology and female fertility (32, 33). Therefore, lipid abnormalities in both partners may reflect a couple-level metabolic burden affecting complementary aspects of gamete function and fertilization potential.

This couple-based perspective is also supported by clinical evidence. The LIFE study showed that lipid concentrations in both male and female partners were associated with couple fecundity and time to pregnancy (34). More recently, Bollig et al. examined preconception lipid abnormalities in both partners among couples seeking fertility treatment and reported associations with live birth outcomes (12). Compared with previous couple-based lipid studies, the present study specifically focused on couples undergoing their first IVF/ICSI cycle and further evaluated embryological outcomes, cumulative miscarriage, and cumulative live birth derived from the same ovarian stimulation cycle. Although the present analysis was not designed to formally test statistical interaction, the less favorable outcomes observed in couples with bilateral dyslipidemia may indicate a possible couple-level association pattern. Given the relatively small number of couples with bilateral dyslipidemia and the limited number of cumulative miscarriage events, this finding should be interpreted as exploratory and requires further validation in larger prospective cohorts.

The RCS analyses were used to further explore potential association patterns across lipid levels. For cumulative miscarriage, female TC, female LDL-C, and male TC showed significant overall associations, whereas the tests for nonlinearity were not statistically significant, suggesting approximately linear association patterns. For cumulative live birth, female TG showed a significant nonlinear association, and male TC showed an approximately linear inverse association. However, given the limited sample size and event numbers, these RCS findings should be interpreted as exploratory rather than confirmatory. Larger studies are needed to determine whether these patterns are reproducible across different populations.

Another issue that should be considered is the heterogeneity of dyslipidemia phenotypes. In the present study, dyslipidemia was defined using a composite criterion based on elevated TC, elevated TG, elevated LDL-C, or low HDL-C. The descriptive lipid profile analysis showed that dyslipidemic participants did not represent a uniform metabolic subgroup but included individuals with single lipid abnormalities as well as those with multiple lipid abnormalities. This pattern was particularly evident among male partners and couples with bilateral dyslipidemia, in whom multiple lipid abnormalities were relatively common. Therefore, the observed associations should be interpreted as reflecting an overall dyslipidemic metabolic profile rather than the effect of a single lipid component. These phenotypic differences may have distinct biological implications and could partly explain the variability in associations observed across specific lipid parameters and reproductive outcomes.

## 5 Limitations

Several limitations should be acknowledged. First, this was a single-center retrospective cohort study with a relatively limited sample size, which may restrict causal inference and generalizability. Second, BMI differed significantly between dyslipidemic and normolipidemic groups and may represent an important confounder, because adiposity is closely related to lipid metabolism and ART outcomes. Although the multivariable models were adjusted for female BMI category and male BMI, BMI and lipid metabolism are closely interrelated, and residual confounding related to adiposity, insulin resistance, metabolic syndrome, lifestyle, diet, and other unmeasured metabolic factors cannot be fully excluded. Third, the number of cumulative miscarriage events was limited, with only 32 miscarriage events among couples who achieved cumulative clinical pregnancy. This limitation may affect the stability of multivariable models, particularly in couple-level and RCS analyses, and may partly explain the wide confidence intervals observed in some estimates. Therefore, the findings related to cumulative miscarriage, especially those for bilateral dyslipidemia, should be interpreted cautiously. Fourth, dyslipidemia was defined using a composite criterion, and different lipid phenotypes or combinations of lipid abnormalities may have distinct biological implications. Fifth, ovarian stimulation protocols were individualized in routine clinical practice rather than assigned for research purposes, and this protocol heterogeneity may influence the interpretation of gonadotropin duration and total gonadotropin dose. Finally, formal interaction testing between female and male dyslipidemia was not performed. In addition, some couples may still have had remaining cryopreserved embryos at the time of data extraction, and therefore cumulative live birth outcomes may have been underestimated.

## 6 Conclusion

Dyslipidemia in either or both partners was associated with less favorable embryological and cumulative pregnancy outcomes in IVF/ICSI cycles. Couples with bilateral dyslipidemia appeared to show less favorable outcome patterns, but these findings should be interpreted cautiously given the retrospective design, limited sample size, limited cumulative miscarriage events, and potential residual confounding. Larger prospective studies are warranted to validate these findings and to further clarify the clinical relevance of preconception lipid status in both partners before ART treatment.

## Data Availability

The datasets generated and/or analyzed during the current study are not publicly available due to patient confidentiality and institutional regulations but are available from the corresponding author on reasonable request. Requests to access the datasets should be directed to Weihong Li, liweihong1211@163.com.
