# Supplementary material for: Couple-level dyslipidemia and embryological and cumulative pregnancy outcomes in IVF/ICSI cycles: a single-center retrospective cohort study
Source: Front Endocrinol (Lausanne). 2026 Jun 18;17:1796100. doi: 10.3389/fendo.2026.1796100 (PMC13322922; doi:10.3389/fendo.2026.1796100)
Supplement: Supplementary file 1 [file DataSheet1.pdf]

Supplementary Table S1. Serum lipid profiles and dyslipidemia phenotypes according to dyslipidemia status

Panel A. Lipid profiles according to female dyslipidemia status

Variable	NLF, n=144	DYSLIP-F, n=65	*P* value
Female TC (mmol/L)	4.26 ± 0.50	5.03 ± 0.68	<0.001
Female TG (mmol/L)	0.92 ± 0.30	1.98 ± 1.55	<0.001
Female HDL-C (mmol/L)	1.54 ± 0.30	1.32 ± 0.36	<0.001
Female LDL-C (mmol/L)	2.52 ± 0.46	3.16 ± 0.74	<0.001
Elevated TC, n/N (%)	0/144 (0.00%)	31/65 (47.69%)	-
Elevated TG, n/N (%)	0/144 (0.00%)	34/65 (52.31%)	-
Elevated LDL-C, n/N (%)	0/144 (0.00%)	26/65 (40.00%)	-
Low HDL-C, n/N (%)	0/144 (0.00%)	14/65 (21.54%)	-
Single lipid abnormality, n/N (%)	0/144 (0.00%)	32/65 (49.23%)	-
≥2 lipid abnormalities, n/N (%)	0/144 (0.00%)	33/65 (50.77%)	-

Panel B. Lipid profiles according to male dyslipidemia status

Variable	NLM, n=62	DYSLIP-M, n=147	*P* value
Male TC (mmol/L)	4.29 ± 0.54	5.21 ± 0.93	<0.001
Male TG (mmol/L)	1.13 ± 0.32	2.89 ± 3.53	<0.001
Male HDL-C (mmol/L)	1.34 ± 0.23	1.10 ± 0.31	<0.001
Male LDL-C (mmol/L)	2.67 ± 0.47	3.35 ± 0.83	<0.001
Elevated TC, n/N (%)	0/62 (0.00%)	79/147 (53.74%)	-
Elevated TG, n/N (%)	0/62 (0.00%)	101/147 (68.71%)	-
Elevated LDL-C, n/N (%)	0/62 (0.00%)	78/147 (53.06%)	-
Low HDL-C, n/N (%)	0/62 (0.00%)	59/147 (40.14%)	-
Single lipid abnormality, n/N (%)	0/62 (0.00%)	42/147 (28.57%)	-
≥2 lipid abnormalities, n/N (%)	0/62 (0.00%)	105/147 (71.43%)	-

Panel C. Lipid profiles according to couple-level dyslipidemia status

Variable	B-NL, n=37	U-DYS, n=132	B-DYS, n=40	*P* value
Female TC (mmol/L)	4.18 ± 0.53	4.44 ± 0.62	4.97 ± 0.67	<0.001
Female TG (mmol/L)	0.93 ± 0.29	1.06 ± 0.51	2.17 ± 1.88	<0.001
Female HDL-C (mmol/L)	1.49 ± 0.32	1.51 ± 0.32	1.33 ± 0.37	0.008
Female LDL-C (mmol/L)	2.50 ± 0.51	2.68 ± 0.58	3.03 ± 0.82	<0.001
Male TC (mmol/L)	4.21 ± 0.61	5.06 ± 0.94	5.21 ± 0.85	<0.001
Male TG (mmol/L)	1.10 ± 0.30	2.74 ± 3.72	2.30 ± 1.28	<0.001
Male HDL-C (mmol/L)	1.34 ± 0.21	1.14 ± 0.32	1.12 ± 0.31	<0.001
Male LDL-C (mmol/L)	2.59 ± 0.52	3.23 ± 0.79	3.40 ± 0.82	<0.001
Female elevated TC, n/N (%)	0/37 (0.00%)	11/132 (8.33%)	20/40 (50.00%)	-
Female elevated TG, n/N (%)	0/37 (0.00%)	9/132 (6.82%)	25/40 (62.50%)	-
Female elevated LDL-C, n/N (%)	0/37 (0.00%)	11/132 (8.33%)	15/40 (37.50%)	-
Female low HDL-C, n/N (%)	0/37 (0.00%)	5/132 (3.79%)	9/40 (22.50%)	-
Female ≥2 lipid abnormalities, n/N (%)	0/37 (0.00%)	8/132 (6.06%)	25/40 (62.50%)	-
Male elevated TC, n/N (%)	0/37 (0.00%)	56/132 (42.42%)	23/40 (57.50%)	-
Male elevated TG, n/N (%)	0/37 (0.00%)	78/132 (59.09%)	23/40 (57.50%)	-
Male elevated LDL-C, n/N (%)	0/37 (0.00%)	54/132 (40.91%)	24/40 (60.00%)	-
Male low HDL-C, n/N (%)	0/37 (0.00%)	44/132 (33.33%)	15/40 (37.50%)	-
Male ≥2 lipid abnormalities, n/N (%)	0/37 (0.00%)	78/132 (59.09%)	27/40 (67.50%)	-

Note: Continuous variables are presented as mean ± SD, and categorical variables are presented as n/N (%). Elevated TC was defined as TC ≥ 5.2 mmol/L, elevated TG as TG ≥ 1.7 mmol/L, elevated LDL-C as LDL-C ≥ 3.4 mmol/L, and low HDL-C as HDL-C < 1.0 mmol/L. Phenotype categories were not mutually exclusive, except for single lipid abnormality and ≥2 lipid abnormalities. For continuous variables, *P* values were calculated using independent-samples t tests or Mann–Whitney U tests for two-group comparisons and one-way ANOVA or Kruskal–Wallis tests for three-group comparisons, as appropriate. *P* values were not calculated for dyslipidemia phenotype categories because these categories were components of the dyslipidemia definition. B-NL, bilateral normolipidemia; U-DYS, unilateral dyslipidemia; B-DYS, bilateral

dyslipidemia; NLF, normolipidemic female partners; DYSLIP-F, dyslipidemic female partners; NLM, normolipidemic male partners; DYSLIP-M, dyslipidemic male partners; TC, total cholesterol; TG, triglycerides; HDL-C, high-density lipoprotein cholesterol; LDL-C, low-density lipoprotein cholesterol. A two-sided *P* value < 0.05 was considered statistically significant.

Supplementary Table S2. Sensitivity analysis of cumulative live birth after excluding couples with BMI ≥30 kg/m² in either partner

Outcome	Model	Exposure variable	Reference group	aOR	95% CI	*P* value
Cumulative live birth	Partner-level model	Female dyslipidemia	Female normolipidemia	0.309	0.142–0.671	0.003
Cumulative live birth	Partner-level model	Male dyslipidemia	Male normolipidemia	0.302	0.119–0.767	0.012
Cumulative live birth	Couple-level model	Unilateral dyslipidemia	Bilateral normolipidemia	0.361	0.110–1.188	0.094
Cumulative live birth	Couple-level model	Bilateral dyslipidemia	Bilateral normolipidemia	0.101	0.026–0.392	0.001

Note: aOR, adjusted odds ratio; CI, confidence interval; BMI, body mass index. The sensitivity analysis was performed after excluding couples in which either partner had a BMI ≥30 kg/m². A total of 184 couples were included, with 134 cumulative live birth events. The partner-level model included female and male dyslipidemia status simultaneously and was adjusted for female age, female BMI category, male BMI, and cumulative number of embryos transferred. The couple-level model was adjusted for the same covariates, with bilateral normolipidemia as the reference group. A two-sided *P* value < 0.05 was considered statistically significant.
